# Computational Workflow for Genome-Wide DNA Methylation Profiling and Differential Methylation Analysis

**DOI:** 10.21769/BioProtoc.5506

**Published:** 2025-11-05

**Authors:** Pei-Yu Lin, Guan-Jun Lin, Kuan-Lin Chen, Shiang-Chin Huang, Pao-Yang Chen

**Affiliations:** 1 Institute of Plant and Microbial Biology, Academia Sinica, Taipei, Taiwan; 2 Genome and Systems Biology Degree Program, Academia Sinica and National Taiwan University, Taipei, Taiwan; 3 Institute of Plant Biology, National Taiwan University, Taipei, Taiwan

**Keywords:** Bisulfite sequencing, DNA methylation, Bioinformatics pipeline, BS-seq, EM-seq, NGS, DMR, Bioinformatics, CGmap

## Abstract

DNA methylation is a crucial epigenetic modification that influences gene expression and plays a role in various biological processes. High-throughput sequencing techniques, such as bisulfite sequencing (BS-seq) and enzymatic methyl sequencing (EM-seq), enable genome-wide profiling of DNA methylation patterns with single-base resolution. In this protocol, we present a bioinformatics pipeline for analyzing genome-wide DNA methylation. We outline the step-by-step process of the essential analyses, including quality control using FASTQ for BS- and EM-seqs raw reads, read alignment with commonly used aligners such as Bowtie2 and BS-Seeker2, DNA methylation calling to generate CGmap files, identification of differentially methylated regions (DMRs) using tools including MethylC-analyzer and HOME, data visualization, and post-alignment analyses. Compared to existing workflows, this pipeline integrates multiple steps into a single protocol, lowering the technical barrier, improving reproducibility, and offering flexibility for both plant and animal methylome studies. To illustrate the application of BS-seq and EM-seq, we demonstrate a case study on analyzing a mutant in *Arabidopsis thaliana* with a mutation in the *met1* gene, which encodes a DNA methyltransferase, and results in global CG hypomethylation and altered gene regulation. This example highlights the biological insights that can be gained through systematic methylome analysis. Our workflow is adaptable to any organism with a reference genome and provides a robust framework for uncovering methylation-associated regulatory mechanisms. All scripts and detailed instructions are provided in GitHub repository: https://github.com/PaoyangLab/Methylation_Analysis.

Key features

• Provides a comprehensive pipeline for genome-wide DNA methylation analysis.

• Step-by-step command line for DMR identification and post-analysis with visualization.

## Graphical overview


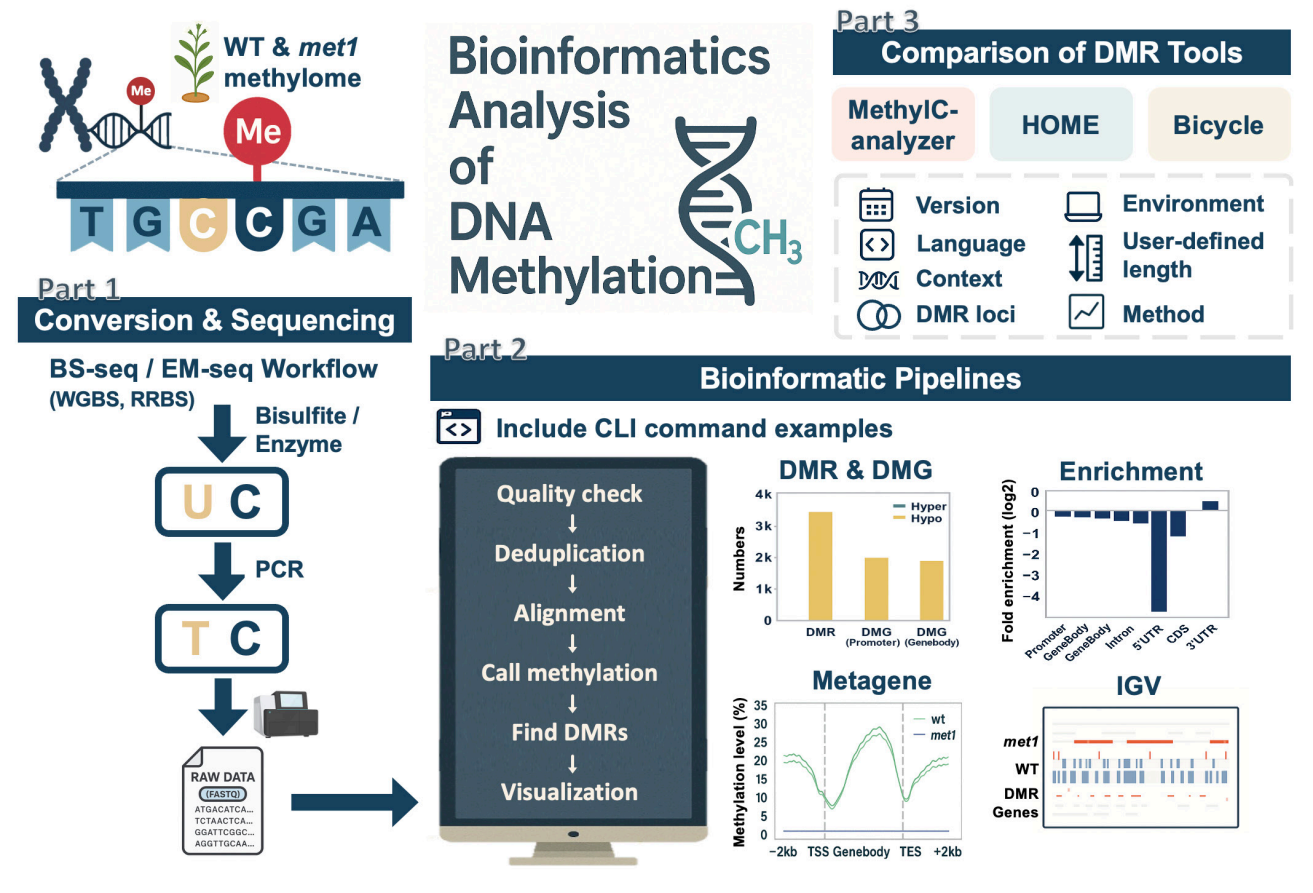


**Overview of bioinformatics analysis of DNA methylation.** An overview of this workflow, including the conversion and sequencing processes of bisulfite sequencing (BS-seq) and enzymatic methyl sequencing (EM-seq), the downstream bioinformatics workflow with example commands, and post-alignment analyses. It includes a comparison of three differentially methylated region (DMR) identification tools, highlighting differences in methodology, feature support, and DMR outputs.

## Background

Epigenetics refers to alterations in gene expression that do not involve any change in the underlying DNA sequences. Such modifications can be inherited and are often reversible [1]. Among all epigenetic factors, DNA methylation is the most studied epigenetic regulator; it refers to the mechanism by which a methyl group is transferred to the C5 position of cytosine to form 5-methylcytosine (5mC) via DNA methyltransferases (DNMTs). DNA methylation occurs in the contexts of symmetric CG and CHG as well as asymmetric CHH sites, where H represents A, C, or T. In symmetric contexts (CG and CHG), methylation can be maintained across DNA replication because the complementary strand provides a template for restoring the methylation pattern. In contrast, methylation at asymmetric CHH sites lacks such symmetry and therefore requires continuous de novo establishment by specific DNA methyltransferases, making it more dynamic and often associated with transposon silencing.

**Figure 1.**
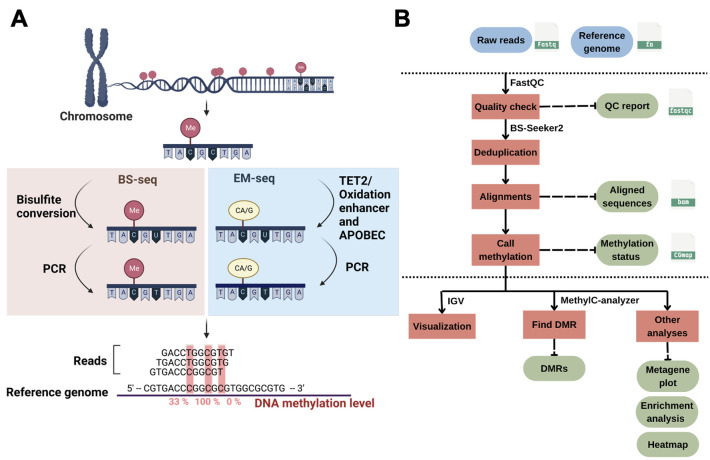
DNA methylation and bioinformatics pipelines overview. (A) Library construction of the enzymatic methyl sequencing (EM-seq) and bisulfite sequencing (BS-seq). Created using BioRender (http://biorender.com/). (B) Workflow of bioinformatics pipelines for DNA methylation analysis. The blue color represents the input data, while the green color is the output. Red boxes are the steps for analysis, and the suggested tools are listed above the box.

DNA methylation can silence genes or transposable elements by changing the chromatin structure or interfering with transcription factor binding [2]. Due to the importance of DNA methylation in biological processes, experimental approaches have been developed to profile genome-wide DNA methylation. Genome-wide DNA methylation profiling is commonly performed using next-generation sequencing (NGS) methods such as reduced-representation bisulfite sequencing (RRBS) [3], whole-genome bisulfite sequencing (WGBS) [4], and enzymatic methyl sequencing (EM-seq) [5]. These NGS-based approaches can determine the methylation status of DNA sequences at single-base resolution and measure DNA methylation levels digitally. In bisulfite sequencing (BS) methods such as RRBS, BS-seq, or WGBS, the crucial step is sodium bisulfite treatment, which converts unmethylated cytosine to uracil (and then to thymine during PCR), while 5mC remains unchanged (**Figure 1A**). Such treatment can result in approximately 84%–96% DNA degradation, causing a significant loss of DNA material and induction of sequence bias, therefore affecting the accuracy of the analyses [6]. To improve from the bisulfite treatment in BS-seq, EM-seq is performed to reduce DNA damage and produce higher-quality libraries for detecting 5mC, and it can generate comparable results using as little as 0.5 ng of input DNA compared with the 200 ng typically required for BS-seq, representing approximately a 400-fold reduction in input material. It uses two sets of enzymatic reactions, methylcytosine dioxygenase 2 (TET2) and T4-phage beta-glucosyltransferase (T4-BGT), to convert 5mC and 5hmC into products that cannot be deaminated by apolipoprotein B mRNA editing enzyme catalytic subunit 3A (APOBEC3A). Then, APOBEC3A deaminates unmodified C to generate U, which is eventually converted into T during PCR (**Figure 1A**) before the final library is sequenced. Compared to BS-seq, EM-seq offers a higher yield and better genome coverage with fewer PCR cycles required [7]. Unlike bisulfite libraries, EM-seq libraries do not exhibit biased AT-rich, GC-poor sequence representation due to the absence of bisulfite treatment–induced DNA damage [5]. Moreover, low-input EM-seq libraries provide similar results to high-input libraries; for instance, a 0.5 ng input of EM-seq covers more CpGs (regions of DNA where a cytosine is followed by a guanine) than the 200 ng input used in BS-seq, highlighting the higher sensitivity of EM-seq [5].

Profiling genome-wide DNA methylation can be computationally intensive [8,9]. The general workflow for such bioinformatics analysis usually includes assessment of read quality, removal of duplicated reads, alignment of reads, quantification of DNA methylation levels, identification of differentially methylated regions (DMRs), visualization of the methylome, and other post-alignment analyses (**Figure 1B**). Each step involves handling large sequencing datasets, often tens to hundreds of gigabytes per sample, which requires substantial computational resources. Alignment of bisulfite-treated reads is particularly demanding due to reduced sequence complexity, and parallel computing is often necessary to achieve reasonable runtimes. Memory usage ranges from 8 to 16 GB for small genomes such as *Arabidopsis thaliana*, but analyses of mammalian WGBS typically require more. Disk space is also a major consideration, with at least 2 TB of available storage being advisable for medium-scale projects.

**Read alignment**

**Figure 2.**
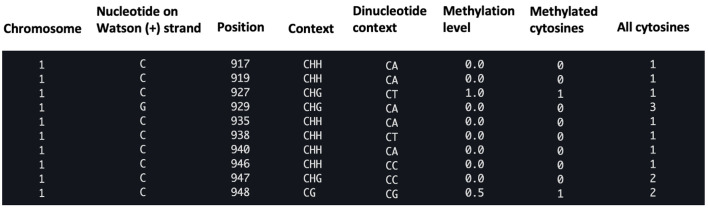
Example of a CGmap file generated with the BS-seeker2 call methylation script. The figure displays a screenshot of the ten rows from the “wt_r1.CGmap.gz” file.

Aligning reads to the reference genome is a critical first step in identifying methylated DNA sites from DNA methylation sequencing data. Commonly used bisulfite-read aligners can carry it out with two types of algorithms: wild-card aligners [10] and three-letter aligners [11]. Wild-card aligners, such as BSMAP [12], replace Cs in the reference genome with the wild-card letter Y, which can match both Cs and Ts in the bisulfite-converted reads. This method offers higher genomic coverage, but can also introduce a bias toward higher methylation levels since it reduces the ability to distinguish between truly methylated cytosines (retained as C) and unmethylated cytosines that have been converted to T. As a result, reads containing Ts may be misaligned to C positions in the reference, leading to an overestimation of methylated sites [11]. On the other hand, three-letter aligners, such as Bismark [13], BS-Seeker2 [14], and BS Seeker3 [15], have higher mapping accuracy but slightly lower coverage compared to the wild-card aligners [11], as they convert all Cs into Ts in the reads for both strands of the sequence, resulting in lower mapping complexity. In general, Bismark is more accurate than BSMAP but its mapping rate and accuracy may decrease with high read error rates in longer reads [16]. BS-Seeker2 is more capable of mapping reads from problematic libraries [14], and is only minimally impacted by read error rates [16]. Overall, among these tools, BSMAP offers the fastest alignment speed and minimal memory usage, while BS-Seeker2 provides the highest mapping accuracy [17]. Also, BS-Seeker3 [15] is an updated version of BS-Seeker2 that enhances alignment accuracy and mappability, while reducing computational time. Common issues during read alignment, like low conversion rate (<98%), may affect accurate cytosine calling and lead to underestimated conversion efficiency. Removing low-quality reads using quality control tools before proceeding can avoid such potential problems. Aligners output the alignments as BAM or SAM files [18] and methylation calling results of each C base as CGmap files [19]; each row contains CpG site information, including chromosome, strand, genomic position, sequence context (CG, CHG, or CHH), dinucleotide context, methylation level, and read counts (**Figure 2**).

Cytosine methylation–level information from CGmap files can be utilized for identifying DMRs. These refer to genomic regions with significantly different levels of DNA methylation between two groups of methylomes—complete sets of DNA methylation patterns across the genome of an organism, cell type, or condition (e.g., experimental and control). The locations of DMRs may be further linked to specific, biologically meaningful features, such as promoters, genes, CpG islands, or other user-defined regions [18,19]. In general, CG sites within promoters often show consistent hypomethylation across biological replicates, correlating with transcriptional activity, whereas intergenic CHH sites are more variable and context-dependent. Additionally, individual variation and potential bias introduced by bisulfite sequencing may contribute to subtle differences between biological replicates, which therefore share similar but not identical methylomes (methylation profiles).

**Differential methylation region (DMR) identification**

Several tools have been developed for DMR detection. Here, we highlight three popular ones, HOME [20], MethylC-analyzer [21], and Bicycle [22] (**Table 1**), each based on different approaches. Machine learning methods make few distributional assumptions and can capture nonlinear patterns. By implementing the machine learning algorithm, HOME utilizes a trained support vector machine (SVM) model to score each cytosine by specific features computed by weighted logistic regression using methylation level differences and p-values between two groups. The tool groups cytosines into DMRs based on scores and distances to their neighboring cytosines [20]. The prebuilt SVM model in HOME has primarily been designed for analyzing mammalian (mainly human) DNA methylation data; therefore, it incorporates assumptions that may not account for the unique genetic regulation in nonmammalian species [23]. DMRs found by HOME are predicted by a precise delineation of the boundaries, and the lengths of the DMRs can vary widely.

Statistical DMR identification tools, such as the MethylC-analyzer, identify DMRs by comparing the average methylation levels (Δ methylation) of the genomic regions between the two groups. A larger difference (e.g., ≥10%) suggests a more substantial shift in epigenetic state. It also offers users a choice between three statistical methods, the Student’s t-test, the Kolmogorov–Smirnov test, and the Mann–Whitney U test, for detecting DMRs with significant differences [21] that reflect that the variation is consistent across replicates, instead of occurring by chance. These statistical methods apply classical hypothesis tests to predefined regions or windows, offering simplicity and speed but inheriting test assumptions (e.g., approximate normality, independent observations) [24] that may be affected when sample sizes are small [25]. Additionally, although users can customize the length of DMRs in MethylC-analyzer, fixed-length regions may still miss irregular DMR boundaries.

As a model-based DMR-finding tool, Bicycle compares methylation levels of user-defined regions between two groups and identifies DMRs using the likelihood ratio test based on beta-binomial models with considerations for sensitivity and specificity [22]. Using beta-binomial models has been claimed to decrease the false-positive rate in DMR identification. In brief, tool selection can be based on data type and analysis requirements, as different tools employ different approaches to define DMRs with diverse lengths and characteristics (see Table 1 for an overview of these tools).

**Table 1.** Comparison of three DMR tools

Feature/tool	HOME	MethylC-analyzer	Bicycle
Version	1.0.0	-	1.8.2
Language	Python, R	Python, R	java
Environment	CLI/	CLI/Docker	CLI
Available context	CG, CHG, CHH	CG, CHG, CHH	CG, CHG, CHH
Testing method	Weighted logistic regression, support vector machine	Student’s t-test, Kolmogorov–Smirnov test, Mann–Whitney U test	Likelihood ratio of beta-binomial models
User-defined DMR length	not available	available	available

CLI: command line interface, GUI: graphical user interface

**Data visualization**

After the reads are aligned, methylome data can be visualized by Integrative Genomic Viewer (IGV) [26] or the UCSC Genome Browser [27]. Users can customize the tracks on both the IGV and UCSC Genome Browser for a better understanding of the global DNA methylation pattern and compare it with other genome features ranging from single-nucleotide to megabase scales. IGV is a user-friendly desktop application that allows users to visualize methylation sites on the genome easily by importing files such as wiggle (WIG) files [27], a text-based format file that stores quantitative genomic data, such as methylation levels or read coverage, across genomic coordinates for efficient visualization. With IGV, we can directly view the methylation levels of identified DMRs and explore the adjacent genomic region that may be the potential regulatory targets of identified DMRs.

**Post-alignment analyses**

Post-alignment analyses aim to associate genomic regions with identified DMRs and explore the roles of these DMRs in genomic regulatory mechanisms, where various toolkits can be applied to such analyses. The R package methylKit [28] can identify DMR proportions in various genetic elements, such as promoters, exons, or enhancers. MethGO [29] provides several modules for analyzing the correlation between methylation level and genomic features, including transcription factor-binding sites (TFBSs). MethylC-analyzer [21] provides an easy-to-use pipeline following the DMR identification step and includes several common analyses, such as enrichment analysis and metagene analysis. Enrichment analysis can assess the preferential localization of DMRs within genomic features across the genome, and metagene analysis is able to show the distribution of methylation levels along the gene body and adjacent regions. MeH is another useful tool that estimates methylation heterogeneity within a population of cells and allows investigation of intra-sample methylation dynamics at specific genomic loci [30].

## Software and datasets

All scripts, examples, and software information (Table 2) are provided in the README at our GitHub repository: https://github.com/PaoyangLab/Methylation_Analysis.

**Table 2.** Required software tools, datasets, and resources used in this pipeline.

Software/dataset/resource	Version	Date	Access		Dependencies	
SRA Toolkit [31]	v3.0.5	May 9, 2023	Open access			
FastQC [32]	v0.12.0	March 1, 2023	Open access		
TrimGalore [33]	v0.6.10	Feb 2, 2023	Open access		
Bowtie2 [34]	v2.26	August 24, 2015	Open access		C/C++ libraries	
BS-Seeker2 [14]	v2.1.8	October 31, 2018	Open access		Python ≥ 2.7	
HOME v1.0.0 [20]	v1.0.0	February 4, 2019	Open access			
MethylC-analyzer [21]	-	January 6, 2023	Open access		Docker ≥ v20.10	
Bicycle [22]	v1.8.2	April 25, 2020	Open access			
IGV Desktop [26]	v2.16.0	April 19, 2023	Open access			
GSE122394 [35]	-	November 20, 2019	Open access			

**Genome-wide DNA methylation dataset**

To demonstrate the methylation analysis pipeline, we downloaded and processed *Arabidopsis thaliana* (GSE122394) BS-seq datasets [35], including wild-type (wt) strains as controls and *met1* mutant strains. The *met1* mutants lack functional DNA methyltransferase 1 (MET1), which is primarily responsible for maintaining CG methylation [36]. Each group contained three biological replicates.

Data for project GSE122394 are available on Gene Expression Omnibus (GEO) and can be accessed using the provided accession codes. The raw reads for each sample are stored in the Sequence Read Archive (SRA) listed in the GEO. To obtain the data, you can use SRAToolkit [33] to download the file using *prefetch* and then convert it into the FASTQ format (.fastq) for analysis by *fast-dump*. A FASTQ file is a text-based format that stores raw sequencing reads along with their corresponding base quality scores from high-throughput sequencing experiments.

*Note:* fasterq-dump *is faster by using multiple threads but requires substantial temporary disk space (approximately 3–4 times the size of the SRA file).*

prefetch SRR8180314 ## download SRA data

fast-dump SRR8180314 ## transfer into fastq file

mv SRR8180314.fastq wt_r1.fastq ## rename the file# Convert SRA file into FASTQ using fasterq-dump (multi-threaded, more efficient)

# fasterq-dump SRR8180314 --split-files -e 8 -t ./tmp

**Hardware requirements**

We recommend a workstation with 8–16 CPU cores, 32–64 GB RAM, and at least 1.5 TB of storage. Raw FASTQ files of *Arabidopsis* typically require 80–120 GB per sample, with BAM and CGmap outputs adding ~40–70 GB each. A dataset of six samples usually needs ~1 TB in total, but allocating ≥2 TB (preferably SSD) ensures smooth analysis.

## Procedure

**A. Processing methylomes**

To provide useful guidance, a bioinformatics pipeline is introduced below, and the tools used in the protocol are listed in the materials section. In the following demonstration, BS-Seeker2 is used.

1. Quality control for methyl-seq reads

a. The methyl-seq reads should undergo quality control (QC) to remove low-quality reads and duplicate sequences generated by PCR amplification and adapter sequences. Recommended tools include FastQC [32] for quality assessment, BS-Seeker2 [14] for duplicate removal, and TrimGalore [33] for reads and adapter trimming. The cutoff for calling low-quality reads is usually set at a Phred score below 20 or 30 [37], which corresponds to an expected base call accuracy of 99%–99.9%. FastQC generates QC reports wt_r1_fastqc.html and wt_r1_fastqc.zip for checking read quality.

# quality control

fastqc wt_r1.fastq

b. Based on the report, the duplicated reads are removed by the *FilterReads.py* from BS-Seeker2, and the adapter and low-quality reads are trimmed by TrimGalore with the new QC report for double-checking. The cleaned and de-duplicated FASTQ files *wt_r1_rmdup_trimmed.fq* are then obtained for the following analysis.

*Note: “-I” specifies the input FASTQ and “-o” is for output FASTQ name. “--fastqc_args” set up the directory of QC report.*

# remove duplicate

./BSseeker2/FilterReads.py -i wt_r1.fastq -o wt_r1_rmdup.fastq > FilterReads.log

# remove adapter

./TrimGalore/trim_galore --fastqc_args "--outdir ./qc_trimming" wt_r1_rmdup.fastq

2. Alignments of methyl-seq reads

a. Download the *Arabidopsis thaliana* TAIR10 reference genome for the aligner.

*Note: The reference genome can be downloaded from iGenomes (* *https://support.illumina.com/sequencing/sequencing_software/igenome.html* *) [38], which offers a collection of reference sequences and annotation files for commonly studied organisms. In this case, the path to downloaded reference genome is: ./Arabidopsis_thaliana/NCBI/TAIR10/Sequence/WholeGenomeFasta/genome.fa*

# download the reference genome

wget https://s3.amazonaws.com/igenomes.illumina.com/Arabidopsis_thaliana/NCBI/TAIR10/Arabidopsis_thaliana_NCBI_TAIR10.tar.gz

tar -xzvf Arabidopsis_thaliana_NCBI_TAIR10.tar.gz ##unzip the file

b. Use Bowtie2 to create a reference genome index file for BS-Seeker2 and save it as “BS2_bt2_Index”. This index generates a set of binary files (e.g., .bt2) that encode the reference genome sequence and its suffix arrays, which are required by the aligner to efficiently map bisulfite- or enzyme-treated reads to the genome.

*Note: The “-f” specifies the FASTA file of the reference genome, and “-d” sets up the directory to save the output files. Path to aligner “-p” will be required if the aligner is in a specific directory.*

# Example commands using BS-Seeker2 (v2.1.8) with Bowtie2 (v2.2.6)

bs_seeker2-build.py -f genome.fa --aligner=bowtie2 -d ./BS2_bt2_Index

c. Align trimmed reads of the wild-type replicate 1 to the reference genome using the align function and save it as a BAM file named “wt_r1_align.bam”.

*Note: “-I” specifies the input FASTQ file and “-o” the output bam file name.*

bs_seeker2-align.py -i wt_r1_rmdup_trimmed.fastq -g genome.fa --aligner=bowtie2 -o wt_r1_align.bam

3. Call methylation.

4. Run the call methylation script to process the aligned BAM file and generate cytosine-level methylation information as a zip CGmap file. The CG map file includes the genomic position, sequence context (CG, CHG, CHH), and the calculated methylation percentage at each site.

*Note: The “-d” parameter is used to specify the index file of the reference genome.*

bs_seeker2-call_methylation.py -i wt_r1_align.bam -o wt_r1.CGmap -d /BS2_bt2_Index/genome.fa_bowtie2

a. View the methylation call output (CGmap). The file with each row represents a single CpG site.

zless wt_r1.CGmap.gz

Each CpG site contains the following information in a row: chromosome, nucleotide on Watson strand, position, context (CG, CHG, or CHH), dinucleotide context, methylation level, number of methylated cytosines (#C), and the total number of all cytosines (#C+T) (**Figure 2**)

5. Conversion rate

For methyl-seq (EM-seq and BS-seq) analysis, it is important to evaluate the conversion rate [39], which measures how effectively bisulfite or enzyme treatment can convert unmethylated cytosines to uracil in DNA samples. This rate can be estimated by comparing the treated genome with an unmethylated bacteriophage lambda genome used as a reference, thereby calculating the percentage of cytosines that were successfully converted. The bacteriophage lambda genome is commonly used as a reference because it is unmethylated under normal conditions, small in size (~48.5 kb), and easy to spike into DNA samples during library preparation. Its lack of endogenous cytosine methylation provides a clean background, allowing accurate estimation of bisulfite or enzymatic conversion efficiency without interference from pre-existing methylation profile. The conversion rate is simply calculated by dividing the number of converted cytosines (#T) by the total number of cytosines (#T+#C) and multiplying by 100, providing the percentage of successful bisulfite or enzymatic conversion. Typically, a conversion rate of 95% or above is preferred because it shows more reliable and accurate results [40].

a. The first step for the conversion rate is the same as above, but changes the input reference genome to the lambda genome.

bs_seeker2-build.py -f lambda_genome.fa --aligner=bowtie2 -d ./BS2_lambda_Index

bs_seeker2-align.py -i wt_r1_rmdup.fastq -g lambda_genome.fa --aligner=bowtie2 -o wt_r1_lambda.bam -m 3 -d BS2_lambda_Index

bs_seeker2-call_methylation.py -i wt_r1_lambda.bam -o wt_r1_lambda -d BS2_lambda_Index/genome.fa_bowtie2/

b. The conversion rate is calculated by the R script with the formula:

$$Conversion\;rate=\frac{\#\;T}{\#\;T+\#\;C}\times 100$$

*Note: The conversion rate script can be viewed or downloaded on the GitHub page: (* *https://github.com/beritlin/NGS_analyses/blob/main/DNA_Methylation_Analyses/coversion_rate.R* *) [41].*

Rscript coversion_rate.R wt_r1_lambda.CGmap.gz

# Output (example):

# Calculating bisulfite conversion rate

# Bisulfite conversion rate: 97.01493 %

In our example, the conversion rate for the wt_r1 methylome is 97.01%, which means that 97.01% of the unmethylated cytosines in the DNA sample have been successfully converted to uracil.

**B. DMR identification**

Here, MethylC-analyzer is selected to demonstrate how to find DMRs from the aligned methylation data output as well as HOME for results comparison. To prevent environmental conflicts, the docker image provided by the software is utilized.

*Note: As different tools require specific environmental settings to run properly, using a docker image can prevent environmental conflict issues.*

1. Searching DMR

**MethylC-analyzer**

a. The command “DMR” is used along with the input “samples_list.txt” file that lists all sample names, CGmap files (wt_r1.CGmap.gz, wt_r2.CGmap.gz, wt_r3.CGmap.gz, met1_r1.CGmap.gz, met1_r2.CGmap.gz, and met1_r3.CGmap.gz, in our case), and the description of each input sample (wt and *met1* in our case), as well as a “gene.gtf” file. The annotation files for *Arabidopsis thaliana* in GTF format can be obtained from the UCSC Genome Browser (https://hgdownload.soe.ucsc.edu/downloads.html); the file contains information about genomic features of genes, such as exons, introns, coding sequences, and untranslated regions (UTRs) [7]. By default, the minimum read depth for each CpG site “-d” and the minimum number of qualified sites within each region “-q” are both set to four. The default region size for DMR searching “-r” is 500 base pairs (bp). To identify DMR, statistical significance is analyzed by Student’s t-test using a default cutoff “-pvalue” of 0.05, and regions must also show an absolute average methylation difference “-dmrc” higher than 10%. All these arguments can be adjusted by users.

*Note: The text in the input file should be separated by a tab. “-a” and “-b” are the group names in the input text file.*

# create input file

vim samples_list.txt

# sample name \t file name \t group name

wt1 wt1.CGmap.gz WT

wt2 wt2.CGmap.gz WT

wt3 wt3.CGmap.gz WT

met1_1 met1_1.CGmap.gz met1

met1_2 met1_2.CGmap.gz met1

met1_3 met1_3.CGmap.gz met1

# run methylc-analyzer

docker run --rm -v $(pwd):/app peiyulin/methylc:V1.0 python /MethylC-analyzer/scripts/MethylC.py DMR samples_list.txt gene.gtf /app/-a met1 -b wt

b. There are three output files: DMR_CG_all_0.1.txt, DMR_CG_hyper_0.1.txt, and DMR_CG_hypo_0.1.txt. These consist of all, hyper, and hypo DMRs with their locations, ∆ methylation, and p-value between groups. Here, we found 3,282 DMRs in CG methylation between the wt and *met1* groups. These DMRs are the genomic regions that show significantly lower methylation levels when MET1 is mutated, indicating that the disruption of this DNA methyltransferase leads to widespread CG hypomethylation; this change may alter the epigenetic regulation of gene expression.

**HOME**

a. HOME requires the input file “sample_file.tsv” with the group name (e.g., wt and *met1*) in the first column and all CGmap files for each group in the second column, separated by a tab. It can only analyze one specific context at a time during the process. The minimum number of sites “-mc” within a region is set as four to match the MethylC-analyzer. By default, the minimum size “-ml” of a DMR is 50 bp, and the minimum average difference “-d” in methylation required in a DMR is 10%. DMR detection is based on a machine learning framework that integrates weighted logistic regression and support vector machine (SVM) classification. The classifier score cutoff “-sc” and pruning cutoff “-p” are both set to 0.1 by default. The corresponding option is specified when using CGmap files generated from BS-Seeker2 directly to ensure compatibility. Arguments can be modified by the users.

# create input file

vim sample_file.tsv

# group name \t file name \t …

wt wt1.CGmap.gz wt2.CGmap.gz wt3.CGmap.gz

met1 met1_1.CGmap.gz met1_2.CGmap.gz met1_3.CGmap.gz

# run HOME

HOME-pairwise -t CG -i sample_file.tsv -o ./-mc 4 --BSSeeker2

b. The output consists of DMR text files for each chromosome “HOME_DMRs_1.txt”, “HOME_DMRs_2.txt”, and so on. In total, 16,185 hypomethylated CG DMRs were detected between the wt and *met1* samples.

2. Analyzing DMR

**Figure 3.**
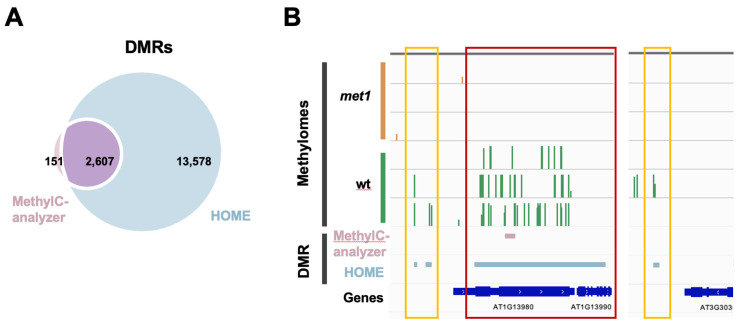
Differentially methylated regions (DMRs) found by MethylC-analyzer and HOME. (A) Venn diagram showing the number of overlapping DMRs between HOME and MethylC-analyzer. The criteria for DMR identification were a minimum of four cytosines within a DMR, a ∆ methylation level cutoff = 0.1, and a p-value < 0.05. (B) Comparison of identified DMRs between HOME and MethylC-analyzer in IGV. The cross-gene DMR is highlighted in red, and the intergenic DMRs are in yellow.

The results of DMR identification tools are compared and shown in **Figure 3**. In total, MethylC-analyzer discovered 3,282 DMRs in fixed regions of 500 bp, which were subsequently merged into 2,785 DMRs by combining contiguous DMRs (size range from 500 to 14,500 bp) in order to avoid fragmentation and to better capture extended regions of differential methylation. HOME identified 16,185 DMRs in regions of varying lengths, with the longest being 36,721 bp and the shortest being 50 bp. HOME identified more DMRs and covered 94.5% of the DMRs found by MethylC-analyzer (**Figure 3A**). Moreover, it can be observed that the DMRs identified by HOME are much wider and span a large region, even extending across multiple genes (**Figure 3B** red box), and those small-sized DMRs tend to spread out in the intergenic regions (IGR) (**Figure 3B** yellow boxes). To sum up, HOME identifies more DMRs than MethylC-analyzer, while HOME is more sensitive to the changes between two groups, and MethylC-analyzer may be more precise by pinpointing the smaller regions.

**C. Data visualization**

1. Genome browser

a. Download and activate the IGV Desktop application according to the operating system (see General note 2). This application supports operating systems including macOS, Windows, and Linux.

b. Select the reference genome from the dropdown list. Here, we chose *A. thaliana* (TAIR10) as a reference genome. Additional reference genomes can be downloaded by clicking *More* or loaded from the local path (in FASTA format).

c. Convert the file from the WIG file to the suggested track formats, BigWig [42] or TDF files, by running IGVtools (click *Tools* > *Run IGVtools*).

*Note: The BigWig file can be obtained by either MethylC-analyzer “metaplot” (see Section D2a) or deepTools “bamCoverage” function from BAM file “-b” and generate output “-o” BIGWIG track file.*

# deepTools

**Figure 4.**
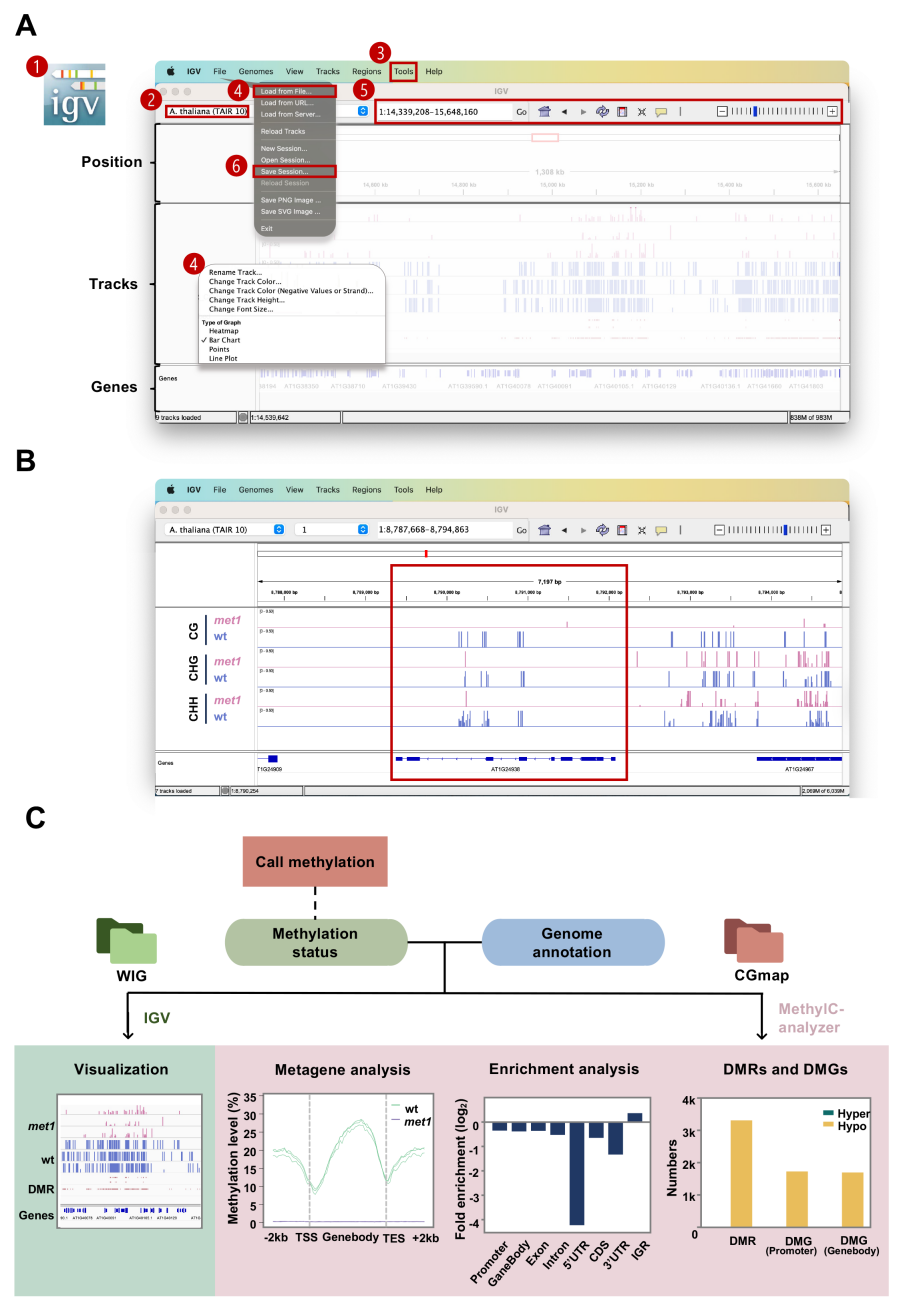
Schematic for post-alignment analysis and visualization. (A) Interface of the IGV desktop on a Mac system. The steps and operating areas are in red (see steps C1a–f). The main steps shown in the figure are as follows: 1) open IGV; 2) select the reference genome; 3) convert WIG to BigWig (*Tools > Run IGVtools*); 4) load tracks (*File > Load from File*) and adjust tracks; 5) select the region of interest; 6) save session (*File > Save session*). (B) Screenshot for IGV. (C) Overview of the post-alignment analyses. Analyses in the right panel (pink) are performed by the MethylC-analyzer, which requires a CGmap and a genome annotation GTF file as input. Visualization by IGV is in the left panel (green). It allows the WIG file from aligners, as well as BigWig and BED files from MethylC-analyzer.

bamCoverage -b wt_r1_align.bam -o wt_r1.bw

d. Select *File* > *Load* to load data into the track panel. Right-click the panel to adjust the graphic type or other settings.

e. Use the dropdown list and search box at the top panel to select the chromosome and region shown. Click *+/-* on the top panel to zoom in/out. Clicking or dragging on the track of the chromosome also adjusts the region shown.

f. Click *File* > *Save session* or *File* > *Save Image* to save the visualization result (**Figure 4A**).

In our example, within the *AT1G24938* locus (a transposable element gene), *met1* samples exhibit lower methylation levels across all contexts compared to wt, with the largest reduction observed in the CG context. This observation is consistent with MET1’s role in maintaining CG methylation (**Figure 4B**).

**D. Post-alignment analyses**

For better interpretation of methylation data, post-alignment analyses like enrichment analysis or metagene analysis are commonly carried out to explain the methylation profiles. Enrichment analysis calculates the fold change in genomic region enrichment in identified DMRs compared to the whole genome. Metagene analysis represents the average methylation level along the gene body and adjacent regions at a normalized length. In this section, MethylC-analyzer is applied to perform enrichment and metagene analyses.

*Note: MethylC-analyzer provides an all-in-one process to perform multiple analyses for the same dataset in one command to save running time.*

1. Enrichment analysis

a. Use the “Fold_Enrichment” command to generate the enrichment result with the “samples_list.txt” file as in step B1a. This module generates output files, including “CG_Fold_Enrichment.pdf” and multiple BED files, such as “CommonRegion_CG.txt.bed”. The BED format provides information like the positions of common methylated regions across samples. The BED file can be visualized by using IGV.

docker run --rm -v $(pwd):/app peiyulin/methylc:V1.0 python /MethylC-analyzer/scripts/MethylC.py Fold_Enrichment samples_list.txt gene.gtf /app/-a met1 -b wt

DMRs exhibit a positive fold enrichment value in the IGR, suggesting a higher likelihood of DMRs being located in IGRs (**Figure 4C**).

2. Metagene analysis

a. Use the “Metaplot” command to generate the Metaplot result. This module generates two types of metagene plots: one represents the average methylation level in two groups (metaplot_CG.pdf), and the other shows the difference between the two groups (metaplot_delta_CG.pdf). The former illustrates the methylation pattern along the gene body and adjacent region, while the latter directly represents the difference in distribution between wt and *met1*. This module also generates BigWig files (met1_r1_CG.bw) to record methylated C sites in metagene analysis, and these BigWig files can be visualized by IGV.

docker run --rm -v $(pwd):/app peiyulin/methylc:V1.0 python /MethylC-analyzer/scripts/MethylC.py Metaplot samples_list.txt gene.gtf /app/-a met1 -b mt

In our case, the wt samples exhibit a standard CG methylation pattern [7] with a lower methylation level at the transcription start site (TSS) and transcription end site (TES). The *met1* samples show a consistently low methylation level along the gene body, reflecting the dysfunction of the methyltransferase (**Figure 4C**).

3. Differentially methylated genes (DMG) analysis

a. Use the “DMG” command to identify those genes or promoters containing DMRs. This module generates output files, including txt files and bed files for the hypo- and hyper-DMRs and DMGs.

docker run --rm -v $(pwd):/app peiyulin/methylc:V1.0 python /MethylC-analyzer/scripts/MethylC.py DMG samples_list.txt gene.gtf /app/-a met1 -b wt

The number of DMRs found in the previous step and DMGs are shown in a bar chart in **Figure 4C**. DMRs in promoters are typically associated with transcriptional activation or repression, while those in gene bodies relate to transcriptional stability and splicing. Similar numbers in both DMGs suggest that methylation changes have no preference between the promoter or the gene body.

## Validation of protocol

This protocol has been used and validated in several plant and human studies. Approaches from read alignment to methylation calling were applied in Hsieh et al. [43,44], Lin et al. [30], Hossain et al. [45], and Lu et al. [21]. The DMR identification step was also used in Hsieh et al. [43,44] and Lin et al. [30] with different criteria.

This protocol (or parts of it) has been used and validated in the following research articles:

- Hsieh et al. [43]. Epigenetic factors direct synergistic and antagonistic regulation of transposable elements in Arabidopsis. *Plant Physiology* (Figure 2E).
- Lin et al. [30]. Estimating genome-wide DNA methylation heterogeneity with methylation patterns. *Epigenetics & Chromatin* (Figures 2, 3, and 4).
- Hsieh et al. [44]. Rice transformation treatments leave specific epigenome changes beyond tissue culture. *Plant Physiology*.
- Lu et al. [21]. MethylC-analyzer: a comprehensive downstream pipeline for the analysis of genome-wide DNA methylation. *Botanical Studies*.

## General notes and troubleshooting

**General notes**

1. The UCSC Genome Browser provides web-based track hubs, which are convenient for users to quickly find and visualize public genome-wide datasets. Users who are looking for more detailed genomic information on well-studied genomes (e.g., the human genome hg38) are recommended to use the UCSC Genome Browser for visualization.

2. In the comparison of different DMR identifiers, we only discussed the difference between HOME and MethylC-analyzer since Bicycle requires its specific file format from its own pipeline. For a fair comparison, the regions of DMR require at least four Cs when applying both tools.

**Troubleshooting**

Problem 1: Low conversion rate (below 98%).

Possible cause: Contamination may affect accurate cytosine calling, leading to underestimated conversion efficiency.

Solution: Remove low-quality reads using quality control tools (e.g., FastQC + Trim Galore) before data analysis.

Problem 2: Low genome coverage leads to biased results.

Possible cause: Insufficient sequencing depth or uneven read distribution across the genome.

Solution: Set a minimum coverage cutoff (e.g., depth ≥ 4 or higher) during analysis to reduce noise and improve reliability.
